# Antisense-Mediated Knockdown of Na_V_1.8, but Not Na_V_1.9, Generates Inhibitory Effects on Complete Freund's Adjuvant-Induced Inflammatory Pain in Rat

**DOI:** 10.1371/journal.pone.0019865

**Published:** 2011-05-10

**Authors:** Yao-Qing Yu, Feng Zhao, Su-Min Guan, Jun Chen

**Affiliations:** 1 Institute for Biomedical Sciences of Pain and Institute for Functional Brain Disorders, Tangdu Hospital, the Fourth Military Medical University, Xi'an, People's Republic of China; 2 School of Stomatology, the Fourth Military Medical University, Xi'an, People's Republic of China; 3 Institute for Biomedical Sciences of Pain, Capital Medical University, Beijing, People's Republic of China; Southern Illinois University School of Medicine, United States of America

## Abstract

Tetrodotoxin-resistant (TTX-R) sodium channels Na_V_1.8 and Na_V_1.9 in sensory neurons were known as key pain modulators. Comparing with the widely reported Na_V_1.8, roles of Na_V_1.9 on inflammatory pain are poorly studied by antisense-induced specific gene knockdown. Here, we used molecular, electrophysiological and behavioral methods to examine the effects of antisense oligodeoxynucleotide (AS ODN) targeting Na_V_1.8 and Na_V_1.9 on inflammatory pain. Following complete Freund's adjuvant (CFA) inflammation treatment, Na_V_1.8 and Na_V_1.9 in rat dorsal root ganglion (DRG) up-regulated mRNA and protein expressions and increased sodium current densities. Immunohistochemical data demonstrated that Na_V_1.8 mainly localized in medium and small-sized DRG neurons, whereas Na_V_1.9 only expressed in small-sized DRG neurons. Intrathecal (i.t.) delivery of AS ODN was used to down-regulate Na_V_1.8 or Na_V_1.9 expressions confirmed by immunohistochemistry and western blot. Unexpectedly, behavioral tests showed that only Na_V_1.8 AS ODN, but not Na_V_1.9 AS ODN could reverse CFA-induced heat and mechanical hypersensitivity. Our data indicated that TTX-R sodium channels Na_V_1.8 and Na_V_1.9 in primary sensory neurons played distinct roles in CFA-induced inflammatory pain and suggested that antisense oligodeoxynucleotide-mediated blocking of key pain modulator might point toward a potential treatment strategy against certain types of inflammatory pain.

## Introduction

Chronic inflammatory pain is worldwide medical problem and with only partial or low efficacy treatment options currently available [1]. Investigations have shown that voltage-gated sodium channels, especially tetrodotoxin-resistant (TTX-R) Na_V_1.8 and Na_V_1.9, provided the potential therapeutic targets for inflammatory pain [2]–[5]. At present, several problems have to overcome for the successful application of TTX-R sodium channel blocking drugs. On one hand, Na_V_1.8 or Na_V_1.9 selective blockers with analgesic activity was hard to explore due to the high degree of amino acid sequence homology among the multiple subtypes of VGSCs [6]. Even the selective Na_V_1.8 blocker A-803467 showed some suppressive effects on Na_V_1.2, Na_V_1.3, Na_V_1.5 and Na_V_1.7 at high concentration (over 1 µM) [7]. On the other hand, various old (carbamazepine, phenytoin, lamotrigine and zonisamide) and newly developed (oxcarbazepine, crobenetine) sodium channel blockers also affect other pathological processes such as epilepsy, spasticity, stoke or psychiatry [8], [9]. Therefore, analgesic strategies beyond direct blocking Na_V_1.8 or Na_V_1.9 at protein levels should be considered for inflammatory pain modulation.

Facing to the complex mechanisms of inflammatory pain and little relief of traditional pharmacotherapy, Glorioso et al recently proposed that targeted delivery of specific pain gene therapy to primary afferent neurons will provided novel approach alleviates pain with less systemic side effects or the induction of tolerance [10]. Antisense agents are valuable tools to inhibit the expression of a target gene through mechanical disruption of the translation process and RNase H-mediated RNA degradation [11], [12]. In a recent review, Kurreck compared three types of anti-mRNA strategies including single stranded antisense oligonucleotides, ribozyme-triggered RNA cleavage and small interfering RNA molecules-induced RNA interference. The author pointed that antisense oligonucleotides combined many desired properties such as broad applicability, direct utilization of sequence information, rapid development at low costs, high probability of success and high specificity compared to alternative technologies for gene functionalization and target validation [13]. It was shown that Na_V_1.8 and Na_V_1.9 are preferentially expressed in a subset of primary afferent sensory neurons in dorsal root ganglion (DRG) but not in spinal cord [14], [15]. The special anatomical sites of DRGs lying along the vertebral column by the spine make it available to directly intrathecal delivery antisense agents and minimize potential off-target adverse events [10]. Although the applications of antisense oligodeoxynucleotides (AS ODNs) targeting Na_V_1.8 in some inflammatory pain models have been introduced [16]–[20], data on the effects of Na_V_1.9 AS ODN is very limited.

The other reason triggering our present study came from a recent negative report of Na_V_1.8 and Na_V_1.9 in pathological pain. Leo et al demonstrated that the contributions of Na_V_1.8 and Na_V_1.9 in knockout mice were quite limited and temporarily in multiple models of acute nociception, peripheral inflammation and neuropathic pain [21]. The presumed critical modulations of Na_V_1.8 and Na_V_1.9 on pathological pain seemed to be challenged. Considering the disadvantages of knockout mice in time-consuming, clinical practice, labor intensity and potential developmental compensatory mechanisms, it is necessary to reevaluate the potential roles of Na_V_1.8 and Na_V_1.9 in the process of inflammatory pain by antisense agents at different levels including gene and protein expression, functions and behaviors.

## Results

### Expression profiles of Na_V_1.8 and Na_V_1.9 in DRG neurons under the state of CFA-induced inflammatory pain

**Figure 1:**
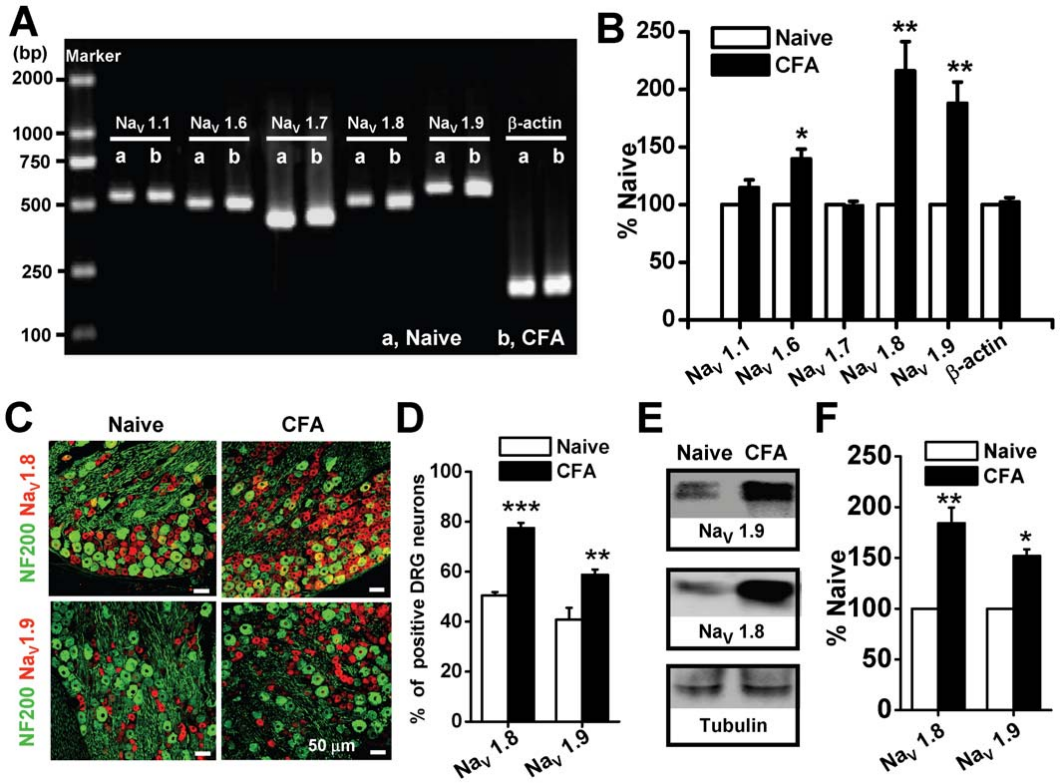
Expression profile of Na_V_1.8 and Na_V_1.9 at mRNA and protein levels following CFA treatment. (A) Representative RT-PCR results of five isoforms of voltage-gated sodium channels. Amplicons of Na_V_1.1, Na_V_1.6, Na_V_1.7, Na_V_1.8, Na_V_1.9 and ß-actin were 540 bp, 509 bp, 441 bp, 515 bp, 572 bp and 229 bp, respectively. (B) Averaged fold changes of mRNA expression as normalized with naive control (n = 3). (C) Double immunofluorescent labeling of DRG neurons by anti-Na_V_1.8 (red) and anti-NF-200 (green) or anti-Na_V_1.9 (red) and anti-NF-200 (green) antibodies. (D) The percentage of Nav1.8- and Nav1.9-positive profiles as a proportion of the total number of DRG neurons before and after CFA treatment (n = 8). (E) Western blotting examples of Na_V_1.8 and Na_V_1.9 in naive and CFA-treated DRG neurons. (F) Averaged protein expression of Na_V_1.8 and Na_V_1.9 between naive and CFA-treated DRGs (normalized with the internal control tubulin) (n = 3 for each group). CFA, complete Freund's adjuvant. * p<0.05, **p<0.01, ***p<0.001.

Following the establishment of CFA-induced inflammatory heat and mechanical pain hypersensitivity (Figure S1), we examined mRNA expressions of fiveisoforms of VGSCs (Na_V_1.1, Na_V_1.6, Na_V_1.7, Na_V_1.8, Na_V_1.9) that mainly expressed in DRG neurons, but not in spinal cord (Figure S2) [15], [22]. DRG were harvested 1 day after CFA injection when the maximum effect on pain hypersensitivity appeared [23], [24]. As shown in Figure 1A and B, there were no changes in the normalized mRNA levels of TTX-S sodium channel Na_V_1.1 (from 100±0.00% to 114.80±6.70%, n = 3, p>0.05) and Na_V_1.7 (from 100±0.00% to 98.99±3.89%, n = 3, p>0.05), except for Na_V_1.6 (from 100±0.00% to 139.63±8.56%, n = 3, p<0.05). The relative mRNA levels of Na_V_1.8 and Na_V_1.9 were significantly increased after CFA inflammation by 216.02±25.52% and 187.86±18.48%, respectively (n = 3, p<0.01).

We further compared Na_V_1.8 and Na_V_1.9 localizations by double immunofluorescent labeling with NF-200, a marker of large- and medium-sized neurons (Figure 1C and D). We found that Na_V_1.8 mainly expressed in medium and small-sized DRG neurons that rarely contain NF-200, whereas Na_V_1.9 just appeared in small-sized DRG neurons that completely not co-localized with NF-200 in naive rats (Figure 1C). After CFA treatment, the percentages of Nav1.8- and Nav1.9-positive stains to the total number of DRG neurons were increased from 50.46±1.39 and 40.78±4.80 to 77.47±2.09 and 58.73±2.15, respectively (Figure 1D, n = 8, p<0.001 or p<0.01). Western blotting showed that the amount of Na_V_1.8 and Na_V_1.9 proteins significantly increased after CFA treatment (Figure 1E). Densitometric analysis revealed that the average expressions of Na_V_1.8 and Na_V_1.9 proteins were increased by 84% and 52% (from 100±0.00 to 184.25±15.37 and 151.97±6.47, respectively. n = 3, p<0.01 or p<0.01) in CFA-treated rats (Figure 1F).

### Enhancement of Na_V_1.8 and Na_V_1.9 sodium currents in DRG neurons following CFA treatment

**Figure 2:**
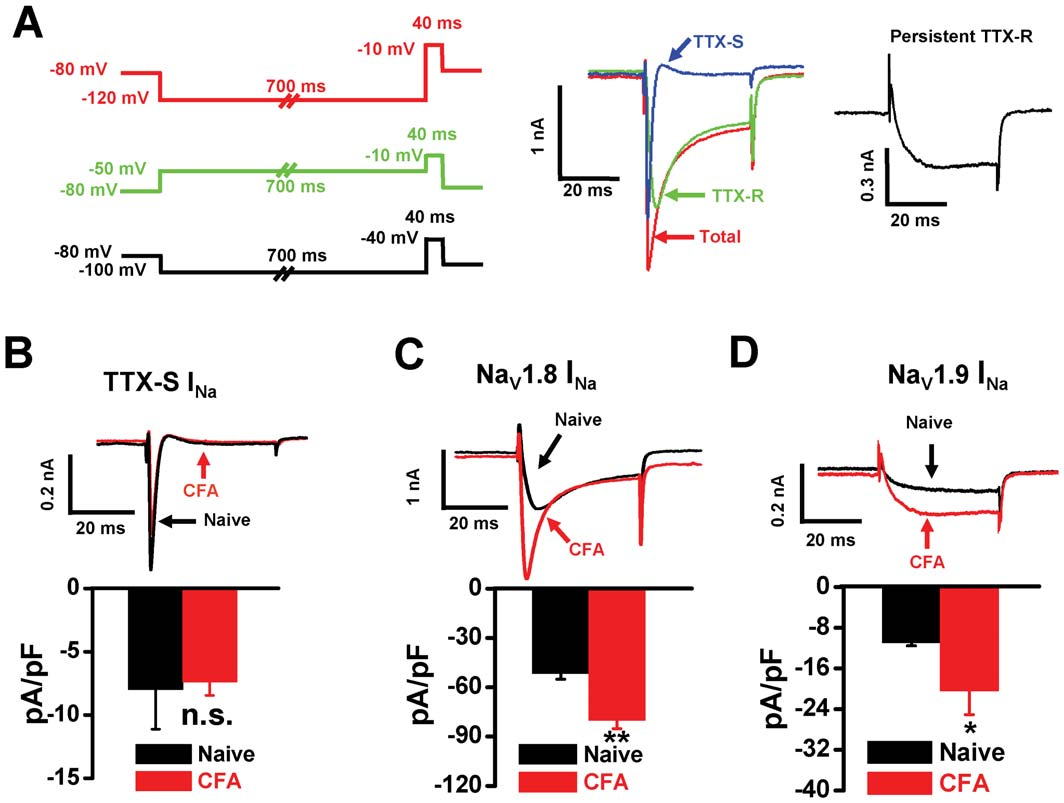
Distinct changes in three components of sodium currents following CFA treatment. (A) Identification of different components of sodium current through electrical stimulus protocols. The total current (red) was recorded with a 700 ms hyperpolarizing prepulse (from −80 mV to −120 mV) followed by a test pulse (40 ms, −10 mV). The TTX-R Na_V_1.8 sodium current (green) was recorded with a 700 ms depolarizing prepulse (from −80 mV to −50 mV) followed by a test pulse (40 ms, −10 mV). The TTX-S sodium current (blue) was obtained by digital subtraction of the TTX-R current from the total current. TTX-R Na_V_1.9 persistent sodium current (black) was recorded with a 700 ms hyperpolarizing prepulse (from −80 mV to −100 mV) followed by a test pulse (40 ms, −40 mV). After CFA treatment, the density of TTX-S sodium current (B) was not changed (n = 6). Whereas the densities of TTX-R sodium currents of Na_V_1.8 (C) and Na_V_1.9 (D) were increased by 55% and 87% (n = 6–8), respectively. CFA, complete Freund's adjuvant. * p<0.05; **p<0.01.

In DRG neurons, we identified three different components of sodium currents, including TTX-S sodium current, Na_V_1.8-mediated TTX-R current and Na_V_1.9-mediated persistent TTX-R current (Figure 2). Electrical parameters for induction of the total current (red) and the TTX-R sodium currents predominantly mediated by Na_V_1.8 (green) and by Na_V_1.9 (black) were shown in the left panel of Figure 2A. The TTX-S sodium current (blue) was obtained by digital subtraction of the TTX-R sodium currents from the total current (middle panel of Figure 2A). After CFA treatment, the density of TTX-S sodium current not changed (Figure 2B, from −7.98±3.13 to −7.37±1.08 pA/pF, n = 8, p>0.05). However, the sodium current densities mediated by Na_V_1.8 and Na_V_1.9 increased 55% (Figure 2C, from −51.61±3.53 to −80.06±5.20 pA/pF, n = 6, p<0.01) and 87% (Figure 2D, from −10.91±0.66 to −20.41±4.70 pA/pF, n = 6–8, p<0.05), respectively.

### Specific down-regulation of Na_V_1.8 and Na_V_1.9 protein expression by i.t. AS ODN

**Figure 3:**
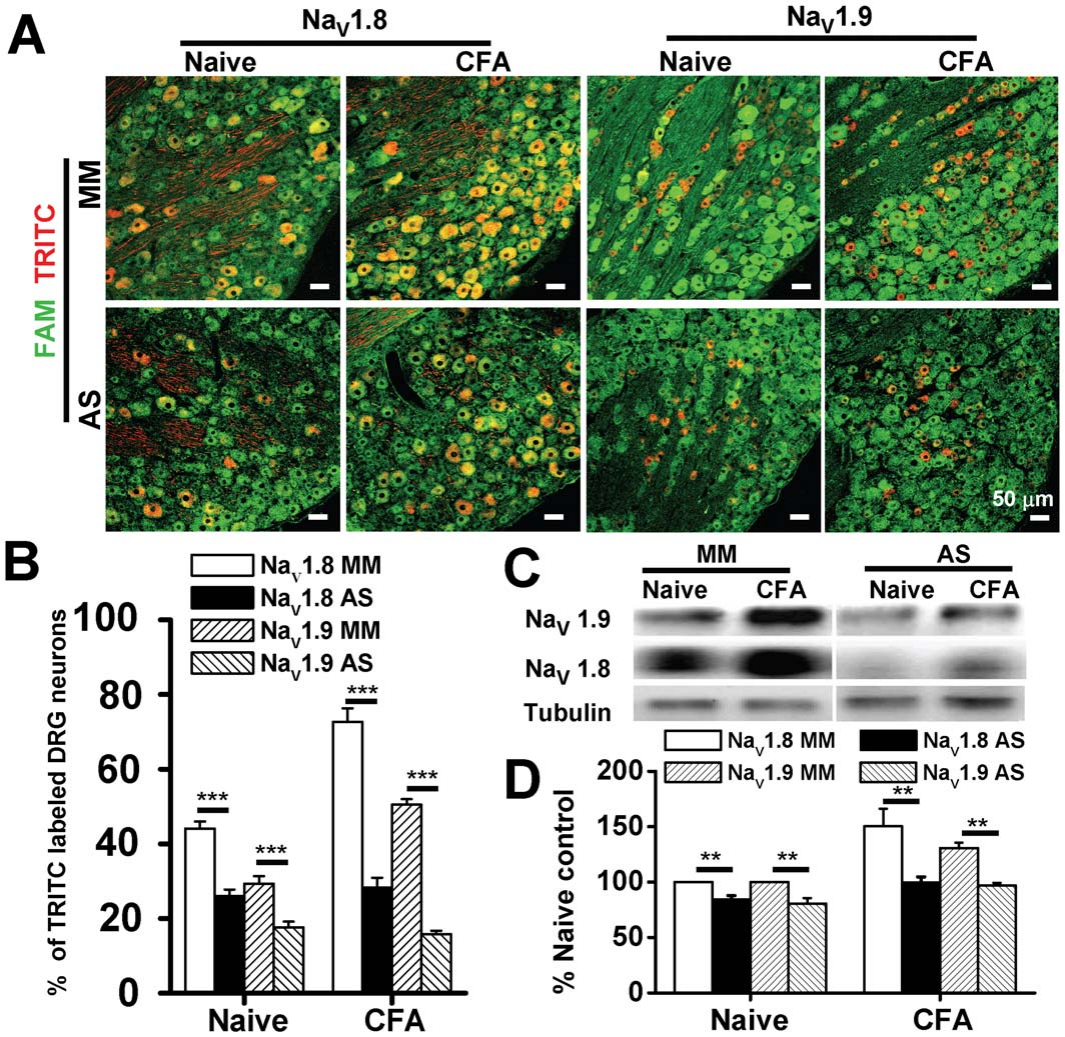
Down-regulation of Na_V_1.8 and Na_V_1.9 expressions in rat DRG neurons by antisense oligodeoxynucleotides (AS ODNs). (A) Examples of double immunofluorescent micrographs of DRG neurons from naive and CFA inflammation. FAM-labeled ODNs (green) were observed primarily in the cytoplasm in nearly all cell bodies of DRG neurons 3 days after i.t. administration of ODNs. (B) The percentage of TRITC-positive stains (Na_V_1.8 or Na_V_1.9) to the total number of FAM-labeled DRG neurons was significantly decreased by AS ODN in both naive and CFA-treated rats (n = 8). (C) Representative western blotting of Na_V_1.8 and Na_V_1.9 in DRG neurons from naive and CFA-inflamed rats following i.t. administration of ODNs. (D) Averaged percent changes in the amount of Na_V_1.8 or Na_V_1.9 protein level in DRGs of naive and CFA-treated rats (n = 5). CFA, complete Freund's adjuvant. AS, antisense. MM, mismatch. ODN, oligodeoxynucleotide. FAM, carboxyfluorescein. TRITC, tetramethylrhodamine-5-(and-6)-isothiocyanate. **p<0.01; ***p<0.001.

Three days after intrathecal (i.t.) administration of FAM-labeled ODNs, expressions of Na_V_1.8 and Nav1.9 were examined by immunohistochemistry. There was no different in the number of FAM-labeled DRG neurons in control and CFA. In control group, the number of FAM-labeled cells in single DRG slice sample were 143.38±10.01, 158.50±5.92, 158.51±6.14, 158.62±8.89 in Na_V_1.8 MM, Na_V_1.8 AS, Na_V_1.9 MM and Na_V_1.9 AS, respectively (n = 8, p>0.05). In CFA group, the number of FAM-labeled cells in single DRG slice sample were 155.50±11.84, 156.13±6.70, 172.38±6.08, 172.50±7.10 in Na_V_1.8 MM, Na_V_1.8 AS, Na_V_1.9 MM and Na_V_1.9 AS, respectively (n = 8, p>0.05). Na_V_1.8 or Na_V_1.9 AS ODN treatment generated marked loss of Na_V_1.8- or Na_V_1.9-positive DRG neurons in both naive and CFA-treated groups (Figure 3A). Compared with MM control, AS ODNs significantly decreased the percentage of TRITC-positive stains (Na_V_1.8 or Na_V_1.9) in naive (Na_V_1.8 MM vs. Na_V_1.8 AS: 44.01±1.88% vs. 26.08±1.70%; Na_V_1.9 MM vs. Na_V_1.9 AS: 29.29±2.05% vs. 17.58±1.55%; n = 8, p<0.001) and CFA-treated rats (Na_V_1.8 MM vs. Na_V_1.8 AS: 72.66±3.68% vs. 28.29±2.61%; Na_V_1.9 MM vs. Na_V_1.9 AS: 50.50±1.49% vs. 15.79±0.90%; n = 8, p<0.001) (Figure 3B). Western blotting further showed that Na_V_1.8 and Na_V_1.9 AS ODNs significantly decreased protein expressions of Na_V_1.8 and Na_V_1.9 in CFA treated rats (Na_V_1.8: from 150.37±15.81% to 99.65±5.04%; Na_V_1.9: from 130.72±4.87% to 96.84±2.49%, n = 5, p<0.01) (Figure 3C and D).

### Inhibitions of Na_V_1.8 and Na_V_1.9 sodium currents by i.t. AS ODN

**Figure 4:**
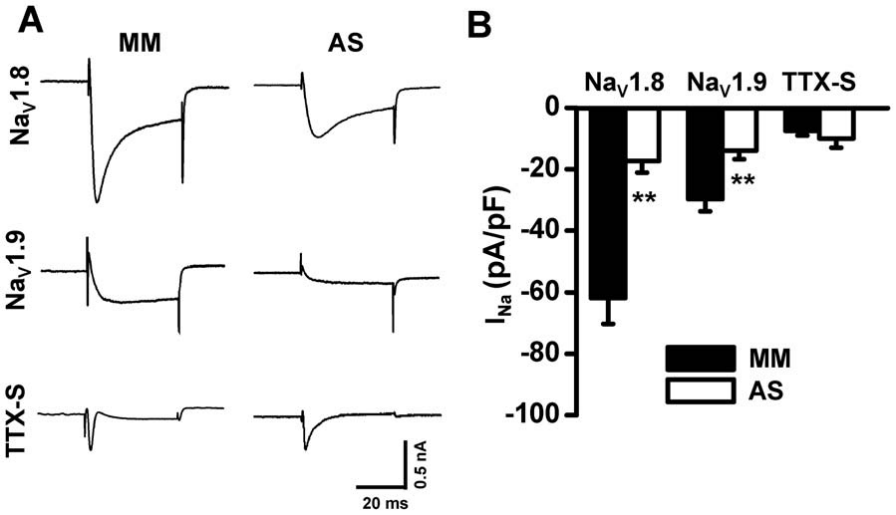
Effects of ODNs on sodium currents in DRGs from CFA-treated rats. (A) Example current traces represent TTX-R sodium channels Na_V_1.8 and Na_V_1.9 and TTX-S sodium channels in DRG neurons 3 days after i.t. administration of MM ODN, Na_V_1.8 AS ODN or Na_V_1.9 AS ODN in CFA-inflamed rats. (B) Averaged sodium current density of Na_V_1.8, Na_V_1.9, and TTX-S sodium currents (n = 6–7) recorded in DRG neurons of CFA-inflamed rats 3 days after i.t. administration of MM ODN, Na_V_1.8 AS ODN or Na_V_1.9 AS ODN. CFA, complete Freund's adjuvant. AS, antisense. MM, mismatch. ODN, oligodeoxynucleotide. **p<0.01.

Three days after i.t. administration of ODNs, the different components of DRG neuronal sodium currents including Na_V_1.8-mediated TTX-R current, Na_V_1.9-mediated persistent TTX-R current and TTX-S current were tested in CFA treated groups (Figure 4A). Compared with MM control, AS ODNs significantly reduced the TTX-R sodium current density of Na_V_1.8 (from −61.79±8.48 to −17.30±3.80 pA/pF, n = 6–7, p<0.01) and Na_V_1.9 (from −29.75±3.97 to −13.93±2.84 pA/pF, n = 6–7, p<0.01). In contrast, AS ODNs had no effects on the TTX-S sodium component (from −7.46±1.55 to −9.96±3.03 pA/pF, n = 6–7, p>0.05) (Figure 4B).

### Reversal effect on CFA-induced pain hypersensitivity by Na_V_1.8, but not Na_V_1.9, AS ODN

**Figure 5:**
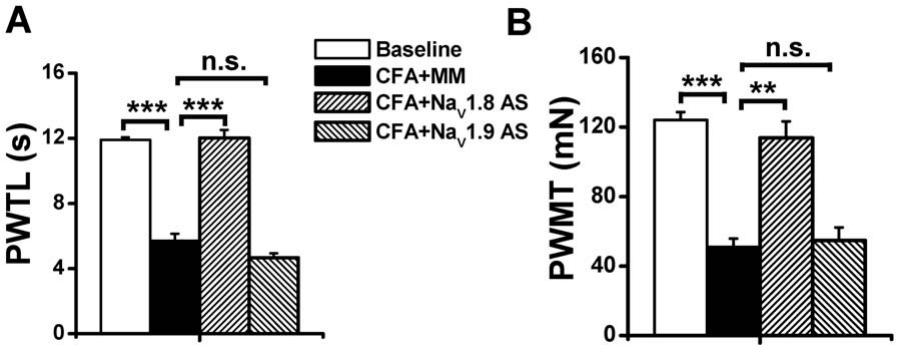
Effects of Na_V_1.8 and Na_V_1.9 ODNs on CFA-induced pain hypersensitivity. MM or AS ODNs (45 µg/5 µl) were i.t. administered twice daily for three consecutive days. CFA was s.c. injected on the morning of day 3 and pain hypersensitivity behaviors were tested on the morning of day 4 afterward while receiving ONDs administration. The results showed that Na_V_1.8 and Na_V_1.9 AS ODNs had different effects on CFA-induced heat (A) and mechanical (B) hypersensitivity. CFA, complete Freund's adjuvant. PWTL, paw withdrawal thermal latency. PWMT, paw withdrawal mechanical threshold. AS, antisense. MM, mismatch. ODN, oligodeoxynucleotide. **, p<0.01; ***, p<0.001.

Compared with the baseline level (11.91±0.16 s), s.c. injection of CFA in MM ODN-treated rats caused significant reduction in PWTL (5.71±0.43 s, n = 5, p<0.001) that could be reversed by Na_V_1.8 AS ODN (12.01±0.49 s, n = 5, p<0.001), but not by Na_V_1.9 AS ODN (4.67±0.27 s, n = 5, p>0.05) (Figure 5A). Similarly, the significant reduction in PWMT of MM ODN-treated rats caused by CFA treatment (from baseline value of 124.13±4.40 mN to 50.96±4.80 mN, n = 5, p<0.001) could be reversed by Na_V_1.8 AS ODN (113.68±9.60 mN, n = 5, p<0.001), but not by Na_V_1.9 AS ODN (54.88±7.33 mN, n = 5, p>0.05) (Figure 5B).

## Discussion

In the present study, we got the following results. First, CFA inflammation treatment caused increased expressions of Na_V_1.8 and Na_V_1.9 in primary sensory DRG neurons. Functional analysis on the different components of voltage-gated sodium currents [25], [26] demonstrated that CFA treatment significantly increased the sodium current densities of Na_V_1.8 and Na_V_1.9, but had no effects on TTX-S sodium currents. Second, intrathecal administration of Na_V_1.8 and Na_V_1.9 AS ODN significantly decreased CFA-induced proteins up-regulation and functional enhancement of TTX-R sodium channels. Third, behavioral tests showed that Na_V_1.8, but not Na_V_1.9, AS ODN could reverse CFA-induced heat and mechanical hypersensitivity. Our data indicated that the key pain modulators Na_V_1.8 and Na_V_1.9 played distinct regulations on the processing of CFA-induced inflammatory pain and that antisense oligodeoxynucleotide-mediated blocking of Na_V_1.8 over-expression might point toward a potential treatment strategy against certain types of pathological pain.

It should be noted that inflammatory pain is complexed with multiple modulators including neurotransmitters, receptors and voltage-gated ion channels [27] and diverse pain behaviors including spinal withdrawal reflexes, spino-bulbospinal jumping or abdominal stretching reflexes and simple vocalization, scratching, biting, licking and guarding behaviors. These pain modulators and behaviors can be evaluated through a variety of pain models (such as formalin, CFA, carrageenan, urate crystals, zymosan, bradykinin, pro-inflammatory cytokines, neurotrophin, prostaglandins, serotonin and substance P in differential degrees [28]. In present study, we reported that Na_V_1.8 antisense generated inhibitory effects on CFA-induced inflammatory pain. However, Na_V_1.8 antisense-mediated inhibitory effects might not generate same biological regulations in all other inflammatory pain models. It was reported that Na_V_1.8 antisense had no effects on carrageenan-induced inflammatory pain behaviors [20]. Our unpublished data also indicated that antisense targeting Na_V_1.9, but not Na_V_1.8 could inhibit pain behaviors induced by melittin, another inflammatory pain model well established in our lab [29]. We think that Na_V_1.8 and Na_V_1.9 exert distinct regulations on inflammatory pain and even the same pain modulator might play different roles in a variety of pain conditions.

Nociceptive DRG neurons expressed TTX-R sodium channels Na_V_1.8 (SNS/PN3) [14] and Na_V_1.9 (NaN/SNS2) [15]. Double labeling studies demonstrated that Na_V_1.9 mainly expressed in non-peptidergic isolectin B4 (IB4)-positive DRG neurons and Na_V_1.8 showed in both IB4-positive and peptidergic IB4-negative and neurons [30]. Na_V_1.8 and Na_V_1.9 have distinct intrinsic ion channel properties related to neuronal functions: (1) Na_V_1.8 and Na_V_1.9 perform different electrogenic properties. Na_V_1.8 has high activation threshold and slow inactivation kinetics and the channel opening leads directly to the generation of action potentials [31], whereas Nav1.9 has a more hyperpolarized activation threshold and generates a prominent persistent sodium current at subthreshold voltages that may regulate subthreshold excitability in small DRG neurons [32], [33]. (2) Na_V_1.8 and Na_V_1.9 contribute differently to AP generation. Na_V_1.8 can modulate AP threshold and overshoot [22] and is responsible for the majority (80–90%) of action potential electrogenesis in nociceptive DRG neurons [34]. However, Na_V_1.9 seems to be not directly involved in the generation of action potentials [35] since neurons from Na_V_1.9 gene knockout mice can exhibit normal action potential characteristics in RMP, threshold, amplitude and duration as shown in wild type mice [36]. These different electrophysiological properties between Na_V_1.8 and Na_V_1.9 might affect the contributions of TTX-R sodium channels in the process of pathological pain.

To our knowledge, the contributions of Na_V_1.9 in pain modulation were poorly understood although some researchers proposed that Na_V_1.9 should play specialized roles in pain pathways due to its restricted distribution within small DRG neurons [15], [37]–[39]. In the current study, we found that although the gene and protein level of Na_V_1.9 were significantly increased and Na_V_1.9-mediated current density was greatly enhanced by CFA treatment, down-regulation of Na_V_1.9 by specific antisense failed to affect CFA-induced pain hypersensitivity. The less contribution of Na_V_1.9 seemed to be consistent with two previous reports. One showed that Na_V_1.9 knockdown had no effect on nerve injury-induced behavioral responses [20], the other showed that Na_V_1.9 knockout failed to change the neuronal hyperexcitability induced by nippostrongylus brasiliensis chronic inflammation [40]. However, another study disrupting Na_V_1.9 coding sequence with IRES-LacZ-neo in mice indicated that the expression of Na_V_1.9 contributed to the persistent heat hypersensitivity after CFA or carrageenan treatment [36]. We noted that, in that paper, CFA-induced thermal latency was not changed in Na_V_1.9 null mice at two time points (6 and 24 h after CFA injection) when the most significant heat hyperalgesic responses appeared in wild type mice. Also, there was no difference in carrageenan-induced thermal latencies between Na_V_1.9 null and wild type mice when heat hyperalgesia was demonstrated at three time points (1, 2 and 3 h after carrageenan injection). Following that report, another gene manipulation experiment by deleting exons 4, 5, and 6 of Na_V_1.9 showed that CFA-induced inflammatory heat hypersensitivity was diminished in Na_V_1.9^−/−^ mice during all the detected time points from 1 to 7 d after inflammation treatment [41]. At present, we don't know why Na_V_1.9 upregulation had nearly no effects on CFA-induced inflammatory pain behaviors. We noted that Na_V_1.9 strictly distributed in small-sized DRG neurons and completely not co-localized with large- and medium-sized neuronal marker NF-200. The limited distribution of Na_V_1.9 in primary sensory neurons might be of no consequence in determining the overall pain level following certain types of inflammation treatment. In addition, different experimental approaches and pain models evaluating Na_V_1.9 should be considered. It was shown that Na_V_1.9 knockout just played inhibitory regulations on imflammation-induced visceral hyperalgesia [42], rather than somatic inflammation and neuropathic pain [21]. Nevertheless, conflicting evidences on the roles of Na_V_1.9 are intriguing for further investigations.

Recently, the potential roles of TTX-S sodium channel Na_V_1.6 were introduced in neuropathic pain models. It was shown that Na_V_1.6 protein significantly increased proximal to the lesion site after infraorbital nerve injury [43] and that Na_V_1.6 gene transcription up-regulated in non-injured L4 DRG neurons following tightly ligation of L5 spinal nerve [44]. In present study, we found that mRNA expression of Na_V_1.6 increased 40% following CFA inflammation treatment. It remains interesting to study whether Na_V_1.6 in DRG participates in inflammatory pain in future. However, providing Na_V_1.6 as the potential analgesic target was quite limited because this sodium channel isoform widely located at nodes of Ranvier of both sensory and motor axons in the peripheral nervous system and at nodes in the central nervous system [45].

As an implantable therapy, intrathecal infusion in the treatment of chronic intractable pain might provide positive long-term outcomes and act as an advanced-stage therapy for refractory pain [46]. In present study, mainly by intrathecal antisense administration protocol, we supported the major contributions of Na_V_1.8 in primary sensory neurons for CFA-induced inflammatory pain behaviors. There is still a long way, however, to succeed in clinical applications since many other factors (such as patient selection, delivery system, potential complication and safety qualification) should be carefully and thoroughly evaluated. Nevertheless, further understanding the pathway, safety, and efficacy of antisense-mediated knockdown of specific pain modulator might point toward a potential treatment strategy against certain types of chronic inflammatory pain.

## Materials and Methods

### Experimental animals

The experiments were performed on male Sprague-Dawley albino rats (purchased from Laboratory Animal Center of the Fourth Military Medical University, FMMU, China) weighing 80–120 g (age >3 postnatal weeks). The animals had access to water and food ad libitum, and maintained at room temperature (22–26°C) with a light/dark cycle of 12 h. The experimental procedures were approved by the Institutional Animal Care and Use Committee at FMMU (approval number: SCXK2007-007). The number of animals used and their sufferings were minimized.

### Behavioral test

CFA (100 µl, 1∶1 dissolved in 0.9% sterile saline) was injected into the plantar surface of rat hindpaws to induce inflammatory pain [47] and maximum effect on pain hypersensitivity appeared 24 h after CFA injection [23], [24]. All behavioral tests were performed by observers blinded to the experimental conditions as previously reported [48], [49]. To assess heat hypersensitivity, rats were placed in a plastic chamber on the surface of a 2 mm thick glass plate and the sensitivity to heat stimuli was detected by RTY-3 radiant heat stimulator (Xi'an Bobang Technologies of Chemical Industry Co. Ltd., China). The heat stimuli were applied to both the injection site and the corresponding area of the contralateral paw, and the latency was determined as the duration from the beginning of heat stimuli to the occurrence of a marked withdrawal reflex. Five stimuli were repeated for each site and the latter three or four values were averaged as mean paw withdrawal thermal latency (PWTL, s). For evaluation of mechanical hypersensitivity, mechanical stimuli were applied by using ascending graded individual von Frey monofilaments with different bending forces (mN): 4.9, 9.8, 19.6, 39.2, 58.8, 78.4, 98.0, 117.6, 137.2, 156.8, 176.4, 196.0, 245.0, 343.0, 441.0, and 588.0. A bending force being able to evoke 50% occurrence of paw withdrawal reflex was expressed as the paw withdrawal mechanical threshold (PWMT, mN).

### Cell preparation

DRGs were prepared to study 1 day after CFA injection when the maximum effect on pain hypersensitivity appeared [23], [24]. Animals were anesthetized with pentobarbital and decapitated, and ganglion was removed and chopped in half. Pieces of ganglia (L4-6) were incubated lasting for 40 min at 37°C with DMEM solution (Sigma-Aldrich, Saint Louis, MO, USA) containing 1 mg/ml collagenase (type I A, Sigma) and 0.4 mg/ml trypsin (type I, Sigma). After three washes in standard external solution (in mM): 150 NaCl, 5 KCl, 1 MgCl_2_, 2.5 CaCl_2_, 10 HEPES and 10 glucose, individual cells were dispersed by trituration with a fire-polished Pasteur pipette and plated on glass cover slips. Cells were incubated in standard external solution at 33°C for 0.5–1 h. Experiments were carried out within 8 h and these cells retained a healthy appearance and had negative resting potentials and overshooting action potentials. We mainly recorded neurons ranging from 20–40 µm in diameter as directly observed through IX71 microscope (Olympus, Japan).

### Whole cell patch-clamp electrophysiology

All recordings were made with EPC10 amplifier and Pulse software (HEKA Elektronik, Germany). The data were analyzed by Igor software (Configuration Metrics, Inc. Oregon, USA). Patch electrodes fabricated with P-97 Puller (Narishige, Japan) had resistances of 3–5 MΩ. After GΩ-seal whole-cell formed at room temperature (20–22°C) under voltage-clamp holding at −70 mV, capacitance transient was cancelled, serious resistance was compensated (>80%) and leak current was subtracted digitally. Initial input resistances were within the range of 800 MΩ to 1.5 GΩ. The liquid junction potential between the pipette solution and the bath solution (approximately −10 mV) was corrected. The membrane capacitance was read from the amplifier to determine the size of cells and to calculate the current density.

For voltage clamp recording of sodium current, electrodes were filled with (in mM): 100 CsCl, 40 tetraethylammonium-Cl, 5 NaCl, 1 CaCl_2_, 2 MgCl_2_, 11 EGTA, 10 HEPES, 2 Mg-ATP (pH 7.2, osmolarity 310 mOsm) [19]. Bath solution used to record whole cell sodium currents contained (in mM): 35 NaCl, 30 tetraethylammonium-Cl, 65 choline-Cl, 0.01 CaCl_2_, 5 MgCl_2_, 10 HEPES, 10 glucose (pH 7.4, osmolarity 320 mOsm). According to previous studies [25], [26], different electrical stimulus protocols were used to clarify the three different components of sodium currents. The total current was recorded with a 700 ms prepulse to −120 mV followed by a test pulse (40 ms, −10 mV). The TTX-R sodium current (predominantly mediated by Na_V_1.8) was recorded with a 700 ms prepulse to −50 mV followed by a test pulse (40 ms, −10 mV). The TTX-S sodium current was obtained by digital subtraction of the TTX-R sodium current from the total current. Pervious studies showed that the maximum current of Na_V_1.9 was generated with depolarizations to about −30 mV [35]. However, the same depolarizations also could activate Na_V_1.8-mediated slow-inactivating current if one DRG neuron expressed both Na_V_1.8 and Na_V_1.9 [50]. To minimize the contamination of Na_V_1.8, we used the following protocol to record persistent Na_V_1.9 current: 700 ms prepulse to −100 mV followed by a test pulse (40 ms, −40 mV) (also see Figure 2a).

### RT-PCR

Total RNA was isolated from L4-L6 DRGs with Total RNA Extraction Kit (Omega Bio-Tek, Norcross, GA, USA) following the manufacturer's instructions. Total RNA (1 µg) was reverse transcribed with BioRT Two Step RT-PCR Kit (BioER, Hangzhou, China) in a 20 µl reaction mixture. Reverse transcription was carried out at 37°C for 10 min, followed by 45°C for 45 min and 95°C for 5 min. Primer sequences specific for rat Na_V_1.1, Na_V_1.6, Na_V_1.7, Na_V_1.8, Na_V_1.9 and ß-actin were based on previously reported (Table S1) [51]. PCR was performed in a 25 µl system containing 2 µl templates, 12.5 µl AmpliTaq (PCR Kit, Amresco, USA) and 0.4 µM primers for each gene on a DNA Thermal Cycler. The PCR conditions were 30 cycles of 94°C for 30 s, 57°C for 30 s, and 72°C for 60 s. PCR products (5 µl) was subjected to electrophoresis on 1.0% agarose gels and stained by ethidium bromide. The stained gels were visualized under UV illumination with FluorChem FC2 (Alpha Innotech Corp., San Leandro, CA, USA) and band intensities were analyzed with AlphaView v.1.3.0 (Alpha Innotech Corp). Three independent PCR reactions were performed to determine the average mRNA change.

### Double immunofluorescent histochemistry

DRGs (L4-6) were fixed with 4% paraformaldehyde in 10 mM phosphate buffer (PB) overnight at 4°C, cytoprotected in 10 mM PB containing 30% sucrose. Transverse frozen sections (20 µm thick) were cut on CM1900 freezing microtome (Leica, Germany), incubated for 4 h in 0.05% Triton X-100 and 10% goat serum in phosphate buffered saline (PBS) at room temperature, followed by incubation with primary antibodies at 4°C overnight with agitation. After three washes with PBS, the sections were incubated with secondary antibodies for 2–3 h at room temperature. The primary antibodies were mouse anti-Neurofilament 200 monoclonal antibody (NF-200, 1∶200, Sigma, USA), rabbit anti-rat Nav1.8 and Nav1.9 antibodies (1∶200, Alomone, Israel). Secondary antibodies were FITC-conjugated bovine anti-mouse IgG (1∶200, Santa Cruz Biotechnology, Inc., CA, USA) and TRITC-conjugated goat anti-rabbit IgG (1∶400, Santa Cruz). Photomicrographic images were obtained under a laser scan confocal fluorescent microscope (Olympus FV1000, Japan). Cells were counted by Image-Pro Plus digitizing software (Olympus, Japan) based on the optical density and the size of the object and only that were clearly positive under the microscope were analyzed.

### Western blotting

Na_V_1.8 and Na_V_1.9 proteins in rat L4-6 DRGs were examined as previously described [52]. Total proteins from rat L4-6 DRGs were extracted by homogenization in ice-cold RIPA lysis buffer (Applygen Technologies Inc., China) containing 50 mM Tris (pH 7.4), 150 mM NaCl, 1% NP-40 and 0.1% sodium dodecyl sulphate (SDS). Protein concentrations were determined by a BCA™ protein assay kit (Thermo Scientific, Rockford, IL, USA.). Samples were heated for 10 min at 95°C with SDS-PAGE sample buffer and same amounts of proteins (30 µg) were separated by 6% SDS-PAGE separation gels, and were subsequently transblotted onto nitrocellulose membranes (Bio-Rad, Hercules, CA, USA). We used rabbit anti-rat Na_V_1.8 and Na_V_1.9 monoclonal antibodies (1∶200, Alomone) as primary antibodies and horseradish peroxidase (HRP)-conjugated goat anti-rabbit IgG as secondary antibody (1∶10000, Bio-Rad). Mouse anti-rat monoclonal ß-tubulin antibody (1∶10000, Sigma-Aldrich) was used as internal control. The membranes were developed with ChemiGlow West chemiluminescent substrate kit (Alpha Innotech Corp) and the signals were captured with FluorChem FC2 (Alpha Innotech Corp.). Scanned images were analyzed by Quantity One 1-D Analysis Software (Bio-Rad).

### Antisense oligodeoxynucleotides delivery

Antisense oligodeoxynucleotides (AS ODNs) specific targeting Na_V_1.8 and Na_V_1.9 were previously reported [20] and the sequences were 5′-TCCTCTGTGCTTGGTTCTGGCCT-3′ and 5′- GCCTTGTCTTTGGACTTCTTC-3′, respectively. The mismatch oligodeoxynucleotide (MM ODN) sequence was 5′-TCCTTCGTGCTGTGTTCGTGCCT-3′. The fluorescence labeling of ODNs were carried out by conjugation of carboxyfluorescein (FAM) to the 5′ end of the ODNs, and synthesized as phosphodiester ODNs using standard O-cyanoethylphosphoramidite chemistry (Shanghai Sanggon Biological Engineering Technology Services Co., Ltd, China). Intrathecal (i.t.) delivery method was previously described [48] and ODNs (45 µg/5 µl, dissolved in nuclease-free ultrapure water) were i.t. administered twice daily for three consecutive days.

### Statiscal analysis

All data were expressed as means ± standard error (SEM). Statistical comparisons were performed using one-way ANOVA followed by Fisher's PLSD test. Statitical significance was indicated by a P value<0.05.

## Supporting Information

Figure S1 CFA-induced inflammatory pain behaviors. Intraplantar injection of complete Freund's adjuvant (CFA) caused significant reduction in PWTL (from 12.71±0.80 to 6.89±1.11 s, n = 6, p<0.05) and PWMT (from 117.60±7.16 to 42.47±9.35 mN, n = 6, p<0.001), suggesting the establishment of CFA-induced inflammatory heat (A) and mechanical (B) pain hypersensitivity.
(TIF)

Figure S2 Comparison of mRNA expressions of α-subunit of voltage-gated sodium channels in DRG and spinal cord. A, By RT-PCR analysis, mRNA encoding Na_V_1.1, Na_V_1.6, Na_V_1.7, Na_V_1.8 and Na_V_1.9 were expressed in DRG. B, mRNA encoding Na_V_1.1, Na_V_1.2, Na_V_1.6 and Na_V_1.7 were shown in spinal cord. It should be noted that mRNA encoding Na_V_1.8 and Na_V_1.9 were undetectable in spinal cord of adult rat.
(TIF)

Table S1 Primers for RT-PCR experiments. Nucleotide sequence of PCR primers was listed with reference to corresponding GenBank accession numbers. For each primer pair, forward and reverse primers were located in different exons to avoid the amplification of genomic DNA.
(DOC)
