# Supplementary figures and images for: Antisense-Mediated Knockdown of NaV1.8, but Not NaV1.9, Generates Inhibitory Effects on Complete Freund's Adjuvant-Induced Inflammatory Pain in Rat
Source: PLoS One. 2011 May 10;6(5):e19865. doi: 10.1371/journal.pone.0019865 (PMC3091880; doi:10.1371/journal.pone.0019865)

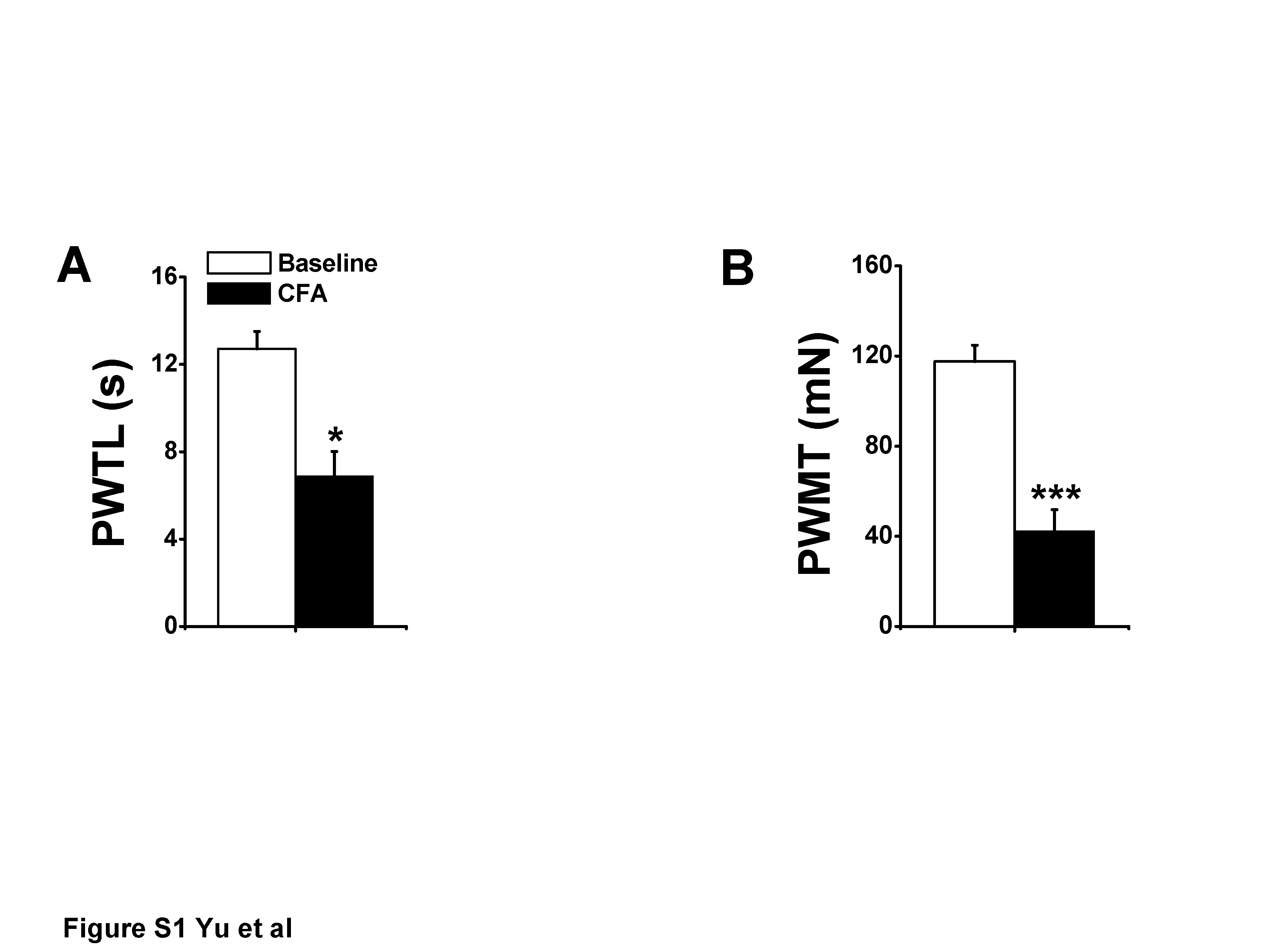

Supplement: Figure S1 — CFA-induced inflammatory pain behaviors. Intraplantar injection of complete Freund's adjuvant (CFA) caused significant reduction in PWTL (from 12.71±0.80 to 6.89±1.11 s, n = 6, p<0.05) and PWMT (from 117.60±7.16 to 42.47±9.35 mN, n = 6, p<0.001), suggesting the establishment of CFA-induced inflammatory heat (A) and mechanical (B) pain hypersensitivity. (TIF) [file pone.0019865.s001.tif]

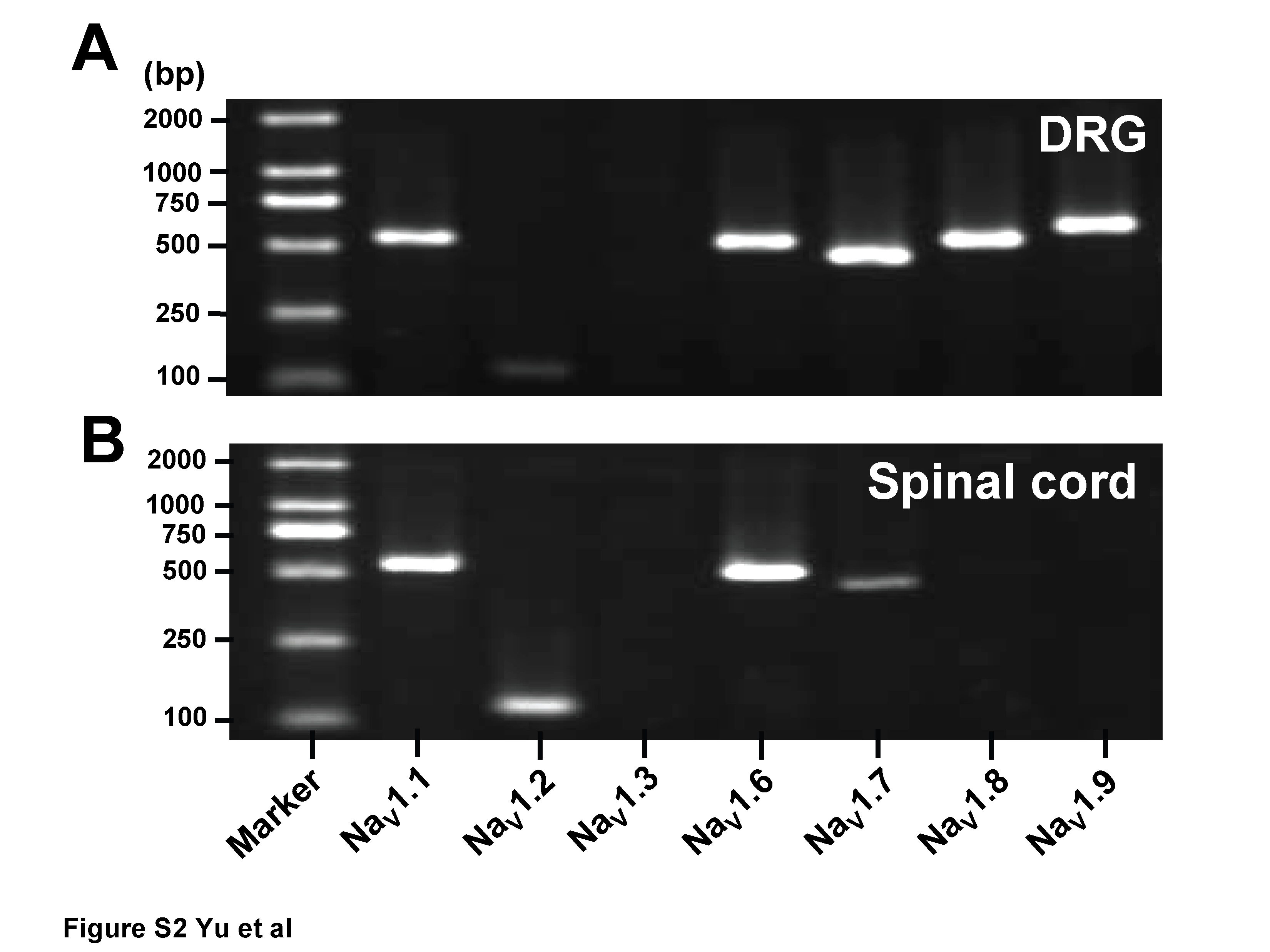

Supplement: Figure S2 — Comparison of mRNA expressions of α-subunit of voltage-gated sodium channels in DRG and spinal cord. A, By RT-PCR analysis, mRNA encoding NaV1.1, NaV1.6, NaV1.7, NaV1.8 and NaV1.9 were expressed in DRG. B, mRNA encoding NaV1.1, NaV1.2, NaV1.6 and NaV1.7 were shown in spinal cord. It should be noted that mRNA encoding NaV1.8 and NaV1.9 were undetectable in spinal cord of adult rat. (TIF) [file pone.0019865.s002.tif]
