# Supplementary material for: Antisense-Mediated Knockdown of NaV1.8, but Not NaV1.9, Generates Inhibitory Effects on Complete Freund's Adjuvant-Induced Inflammatory Pain in Rat
Source: PLoS One. 2011 May 10;6(5):e19865. doi: 10.1371/journal.pone.0019865 (PMC3091880; doi:10.1371/journal.pone.0019865)
Supplement: Table S1 — Primers for RT-PCR experiments. Nucleotide sequence of PCR primers was listed with reference to corresponding GenBank accession numbers. For each primer pair, forward and reverse primers were located in different exons to avoid the amplification of genomic DNA. (DOC) [file pone.0019865.s003.doc]

**Table S1. Primers for RT-PCR experiments**

| **Gene** | **GeneBank accession number** | **Primer sequence** | **Product length (bp)** |
| --- | --- | --- | --- |
| Na_V_1.1 | X03638 | Forward: 5'-GGCGCCACCTTTTGAAGCGA-3'  Reverse: 5'-GCTCTGCACCCCTTTACGCT-3' | 541 |
| Na_V_1.2 | X03639 | Forward: 5'-CATCAAGTCCCTCCGAACGTTA-3'  Reverse: 5'-GGCAGACCAGAAGTACGTTCATT-3' | 131 |
| Na_V_1.3 | Y00766 | Forward: 5'-ATCCGTGTCAACTGGACTCTAAGG-3'  Reverse: 5'-CTTGTGGACTTAGCAACATGGG-3' | 407 |
| Na_V_1.6 | L39018 | Forward: 5'-GCGGGGCTTCATCTGCAGAA-3'  Reverse: 5'-GCCGCCTCGGTTTTGTATCC-3' | 510 |
| Na_V_1.7 | AF000368 | Forward: 5'-GGAGGTGTCCGCGACTATCAT-3'  Reverse: 5'-CTGCCTCGTTGGATCAGAGC-3' | 442 |
| Na_V_1.8 | X92184 | Forward: 5'-CGGGGAGGTGAACAACTTGC-3'  Reverse: 5'-GGGACCTGAGGGCTATCCTT-3' | 516 |
| Na_V_1.9 | AJ237852 | Forward: 5'-CGGGCCTTCTCCTTCATGCT -3'  Reverse: 5' -GACCCAGGGGGACTTTCTCT-3' | 573 |
| β-actin | NM_009073 | Forward:5'-CGTAAAGACCTCTATGCCAACA-3'  Reverse: 5'-CGGACTCATCGTACTCCTGCT-3' | 229 |

Nucleotide sequence of PCR primers is listed with reference to corresponding GenBank accession numbers. For each primer pair, forward and reverse primers are located in different exons to avoid the amplification of genomic DNA.
